# Making a “Happy Hospital”: Emotional Investment and Professional Identity Amongst Anglo-American Hospital Administrators

**DOI:** 10.1093/jhmas/jrad022

**Published:** 2023-05-20

**Authors:** Philip Begley

**Affiliations:** University of Liverpool, UK

**Keywords:** Hospital Administration, National Health Service, US, Britain, Emotions, Training, Healthcare, Work

## Abstract

This article examines the place of emotion in modern hospital administration and the relationship between professional identities and emotional landscapes in the healthcare field. The focus is a broad emotional and philosophical investment that many administrators made in their work. In the United States and then in Britain, amidst rapid change in the practice and provision of health services, a new sense of professional identity emerged. This was often underpinned by a kind of emotional investment, one which had to be constructed and cultivated. Here formal training and education, collective identities, and a shared understanding of the kind of personal qualities required were important. The extent to which developments in Britain were influenced by best practice in the US is also striking. This process might best be understood as the further drawing out of established beliefs and ways of working rather than an abstract transfer of ideas and practices across the Atlantic, but there was a distinct Anglo-American dimension to the development of hospital administration.

In recent years historians of contemporary medicine and health have, just like their modern and early-modern counterparts, become increasingly interested in senses and emotions – as the other articles in this special issue can attest. A number of these studies have looked at the role of emotion in the life and work of doctors, nurses, and other medical professionals.^1^ For example, Agnes Arnold-Forster has begun to reassess the “pervasive and persistent stereotype of the detached surgeon,” one “unable or unwilling to communicate effectively, engage compassionately with their patients, or express how they feel.”^2^ There has also been interest in emotional language as it pertains to patients, and increasingly, to the fabric of the hospital itself – everything from architecture and design to sights, sounds, and smells.^3^ For example, Victoria Bates has discussed the place of patients’ “emotional and holistic needs” in the construction of the hospital environment.^4^ There is one group, however, that has so far been relatively underappreciated by historians – hospital administrators. The aim of this article is to further develop the study of the history of hospital administration, and, in particular, to begin to draw out the place of emotion.

Like other professionals, hospital administrators experienced and expressed emotions in the course of their work – whether frustration at the inefficiency of their colleagues, disgust at the intricacies of the operating theatre, or sympathy for suffering patients. Naturally, this is one of the things that former administrators find easiest to recall:

The first patient was wheeled in soon after, so I had no opportunity of making detailed enquiries, and concentrated solely on the gravitation of my stomach.^5^

A room about this size with thirty-five mentally and physically handicapped people all strapped in chairs, all the way round and in the middle…absolutely awful.^6^

Historians of emotion have shown how such feelings can be understood as individual articulations of innate affects in a socially and culturally constructed setting, as well as the ways in which emotions can help to shape related values and perceptions.^7^ It is easy to imagine how these kinds of experiences might have been formative, and there is real potential for future research in the history of emotions which draws on hospital administration.

The immediate focus of this article however is the broader emotional and philosophical investment that many hospital administrators made in their work, the foundations of which had to be actively constructed and cultivated. Collective approaches such as William M. Reddy’s conception of “emotional regimes” with “mutually comprehensible actors capable of superior cooperation and coordination of effort” are relevant here.^8^ There are synergies with debates about “emotional labour” and the ways in which professional standards and expectations can be used to mobilise feelings.^9^ John Kirk and Christine Wall have also demonstrated the enduring importance of collective experience and expression in the workplace.^10^ In this vein, we can see that from the early decades of the twentieth century, a collective sense of professional identity amongst hospital administrators became increasingly important. This was often underpinned by an emotional investment in the role, and a shared understanding that it required great skill and was crucially important to the successful running of rapidly changing health services. Hospital administration therefore provides an interesting example of the ways in which emotions can be conceptualised and utilised in a healthcare setting. In seeking to understand this dynamic, this article identifies four key thematic points around the importance of the education and training undertaken by administrators: conceptions of the personal qualities required of a good administrator, the reinforcement of collective identities at an institutional level and an individual level, and a striking Anglo-American dimension to the development of modern hospital administration which sought to combine compassionate care and corporate efficiently.

The article begins by tracing the emergence of the modern form of hospital administration in the US during the early decades of the twentieth century. The focus is on senior individuals with responsibility for the successful day-to-day running of the hospital. In the US the key figure was the superintendent appointed by a hospital’s board of trustees. In Britain, the key figures were the medical superintendent (whose role comprised administrative and clinical duties to varying degrees) and the steward in local authority hospitals, and the house governor in voluntary hospitals. After the establishment of the NHS in 1948, depending on their appointment by a board of governors, regional hospital board or hospital management committee, the key figures became group or hospital secretaries, and their deputies and assistants. The ways in which developments in the US influenced those in Britain and the subsequent formalisation and professionalisation of administration, including through the National Administrative Training Scheme from 1956, is then considered. In doing so, the article draws on archival research (principally the records of the Ministry of Health held at the National Archives and those of the King Edward’s Hospital Fund for London held at the London Metropolitan Archives) and oral history interviews (conducted with an experienced set of former NHS administrators). Relevant reflections also emerged during a witness seminar held in 2019 on the work of Professor Teddy Chester of the University of Manchester, a leading figure in administrative training.^11^ Witness seminars bring together a group of invited participants and an expert audience, allowing individual memories to be shared and contested in order to draw out greater insights and construct a fresh analysis of the topic in question.

## Hospital Administration in the US

The development of modern hospital administration can be traced to the late nineteenth and early twentieth centuries and rapid growth in the number of hospitals and hospital activity. J.R. McGibony of the US Public Health Service described this growth as a “development in the health and hospital consciousness of the American public to an extent bordering on the spectacular.”^12^ The concomitant rise of “medical corporatism” and a more complex, technological, and broad-based hospital system helped to create “new layers of hospital administration.”^13^ The defining feature of the new administrative culture was a belief, informed by ideas of scientific management, in the importance of efficiency. The established picture of the hospital administrator for much of the nineteenth century had been one of a caretaker figure with a recognised social standing before entering the field but without formal training or experience. Superintendents were appointed as individuals on the basis of their ability to reflect the moral and religious values of the board of trustees. According to Charles Rosenberg, they often demonstrated the “prudence, responsibility, and piety one might hope for in a business partner or vestryman.”^14^ Amidst changing conditions, however, a new generation of hospital administrators came together and began to organise themselves more distinctly as a modern profession.^15^

Annual meetings of the burgeoning American Hospital Association were particularly important in helping to develop shared knowledge and foster a sense of professional identity.^16^ The early focus was often on the practicalities of being more efficient in the hospital setting, including “how to clean and re-use soiled gauze” and “the proper temperature to store lemons.”^17^ Beyond this, however, administrators could also begin to make good use of their character and emotional intelligence. Rosemary Stevens describes the new administrator as a “harmonizer” – “attempting through his or her personality to command the respect of all participants,” not simply reflecting the values and interests of their own trustees. As such, it was important that they had “tact, good sense, and judgement.”^18^ McGibony also observed that “a difference exists between the ordinary concept of administration and hospital administration. This difference lies largely in the realm of humanitarianism. The successful hospital is not necessarily the one with the most efficient, machine-like business management.”^19^ This evocation of humanity and the human side of hospital administration would be returned to many times. As Rob Boddice has noted, references to humanity often have a “sensory and affective quality” and imply the ability to demonstrate compassion and morality.^20^ It also echoed longstanding debates amongst physicians about the need to address perceived “humanitarian” deficiencies in education and practice – the need for medicine to be an art and a science, and for the clinician to demonstrate “the precision of scientist but also the sensibility of the gentleman.”^21^

In 1942, Raymond P. Sloan, Director of the Modern Hospital in Chicago, outlined the qualities needed by an administrator. In addition to practical knowledge, an administrator should have “a live social conscience,” “a pleasing personality,” “sympathy and understanding,” an “understanding of human nature,” “courage,” “patience,” “loyalty,” a “sense of humor,” and a “sense of fairness.”^22^ Deploying these strengths in the hospital setting would involve a degree of emotional engagement on the part of the administrator, but at the same time would also require “emotional stability.” As Sloan described:

Display of temperament, while unfortunate on the part of any hospital worker, or anyone else for that matter, becomes inexcusable when displayed by the administrator. If he suffers from jumpy nerves, he might better seek surroundings that are less exciting or take definite steps to overcome them. Nervous irritability is a privilege that should be reserved exclusively for the patients and their relatives.^23^

Courage has long been understood in terms of the ability for emotions to be contained and “carried out at the key moment, rather than being experienced passively and beyond ones control.”^24^ The ability to sympathise and demonstrate compassion has also been understood in terms of conveying a sense of shared understanding and commonality to others. The ideal administrator was sincere yet decisive, therefore, and able to demonstrate this to others, but not so emotionally open to patients and fellow staff as to undermine the administrator’s wider role.

One of the ways in which the increasing difficulty and complexity of administration was recognised was the better organised and more formal training programmes which emerged. While earlier administrators had often gained experience in post and undertaken less official preceptorships, the needs of their successors were different. The first meaningful step was a six-month training course for nurse superintendents at Grace Hospital in Detroit in 1910.^25^ After discussion at AHA meetings and more widely over a number of years, the first full graduate course in hospital administration began at the University of Chicago in 1934, under the influence of what Stevens describes as “standard setters” and “leaders of the professionalization movement.”^26^ Steady expansion to meet the growing needs of the sector meant that by 1959, sixteen universities in the US and Canada were offering master’s programmes and a number of others PhD and undergraduate opportunities.

A typical MSc course would last two years and include both practical and theoretical elements. The hospital administration program at Columbia University, for example, included an “academic portion” in which “traditional techniques” were used to cover the principles of hospital organisation and management, and a wide range of subjects in which administrators would need to be well versed – “medical background, hospital law, hospital planning and construction, prepayment of hospital care, biostatistics, elementary epidemiology, public health practice, health education, principle of administration, personnel practices, group processes, and elements of mental health.”^27^ This would be complemented by an “administrative clerkship” – one day per week in a hospital department centred around observation. There was then a full year of “administrative residency” under the supervision of an established administrator. In Chicago this had been known as an “administrative internship,” which helped students to understand “the importance of the human factor in hospital care.”^28^ George F. Billington and his fellow tutors at Columbia saw successful learning as “a private, individual affair which goes on *within* students and depends for its success upon their active participation.”^29^ According to Billington, “The goal of education, broadly stated, is to help students to develop toward effective, independent behavior in their chosen field.”^30^ By the mid to late 1950s, this kind of integrated approach had also become influential across the Atlantic.

## Hospital Administration in Britain

In Britain, administrators had traditionally joined their individual hospital or hospital group at a young age and worked their way up – “the inky-fingers way” – or been recruited with existing professional standing from outside the health field.^31^ Many undertook postal tutorial courses and examinations specific to their voluntary, mental, or local authority hospital, but by the early 1940s there were calls for a more structured set of qualifications.^32^ The foundation of the Institute of Hospital Administrators in 1942 was in part a recognition of this need to supplement “the unsystematised and more or less haphazard method of appointing hospital officers.”^33^ Changing models of hospital finance during the 1930s and 1940s and the centralisation of hospital services that was then on the horizon were also seen to point to the need for greater uniformity and professional recognition.^34^

In terms of organised training, a few apprenticeships had been available at individual hospitals around the country and a more recognised training scheme was organised by the London County Council, though the focus was still on “on the job” training.^35^ After 1945 the King Edward’s Hospital Fund for London (originally founded in 1897 to distribute extra funding amongst London’s voluntary hospitals) began to award bursaries to a small number of administrators who had their careers interrupted by the war, as well as former Army officers. Bursars received an eighteen-month apprenticeship with the house governors of a leading London hospital. However, the most important changes followed the establishment of the NHS in 1948.

That new patterns and costs of providing hospital services would provide further impetus towards professionalisation had been apparent to many observers. For example, Frank Hart and A.J. Waldegrave of the Institute of Public Administration had anticipated the establishment of the NHS as “the prelude to an overhaul of the whole structure and practice of hospital administration.”^36^ They were especially concerned that the “wisdom of established practice” which had been built up over time, particularly in their own voluntary hospitals, might be lost as a result.^37^ This was a philosophical, not just an organisational, question they suggested. There was something – an emotional dimension inherent to the administrators’ role – that it would be important to try and retain:

Administration itself has the features both of a science and an art, and in its practice it needs, like Medicine, however rigorously pursuing scientific efficiency, to preserve the human touch. Its material and resources are the individual men and women who get lumped together under such expressions as staff, personnel and man-power, and on the administrative side as well as on the clinical side there is need to remember always how human an institution the hospital is.^38^

The need to “keep the hospital service *human* – a service preserving human values and not a mere soulless bureaucratic administrative machine” was also noted by Sir George Schuster, Chair of the Oxford Regional Hospital Board.^39^ As in the US, there was overlap with concerns about the lack of a human touch in modern medicine. As Victoria Bates has shown, this debate in Britain played out particularly amongst medical students, and over time there was an increasing recognition that “engagement with the human aspects of medicine was a moral obligation.”^40^

In the event, however, there was no sweeping away of the established administrative approach. Despite the effective nationalisation of hospitals and the introduction of universal provision in 1948, there was a high degree of continuity in terms of personnel and everyday practice. Change was not precipitous. Administrators would naturally be building on what had gone before and it would take time for them to collectively see themselves as part of a new national system. According to C.R. Jolly of the Institute of Hospital Administrators in 1969, “progress in developing new arrangements was hindered – and still remains so – by the fact that the service, although notionally a national one, was administered in England and Wales by more than 400 different hospital authorities, and by another 90 or so authorities in Scotland, each of whom was an autonomous employing authority,” and had a tendency “to live in a world of its own.”^41^ By the late 1940s therefore it appeared as though change, particularly in terms of formal training, would eventually be forthcoming. Nonetheless, at least for an elite group at the top, because of the way that official training schemes then developed, this sense of self amongst hospital administrators and the emotional dimension of their work would actually be drawn out further rather than being squeezed out.

In this regard, an important moment arrived in 1951 when the King’s Fund established its own Hospital Administrative Staff College. It was independent of the NHS but had the support of the Ministry of Health and co-operation from the Institute for Health Administrators who were represented on the managing body. P.H. Constable, House Governor of St George’s Hospital in London, who became the Staff College’s first Director, had visited the US and Canada in 1950. He did so with the assistance of the Kellogg Foundation and the Rockefeller Foundation, who had a longstanding interest in health systems learning across the Atlantic.^42^ Constable was particularly impressed by the programmes offered at the Universities of Toronto, Minnesota, and Columbia, and brought back the conviction that administrators should have a particular aptitude for the work, be able to work well with and serve others – especially the medical staff – and that trainees were best served by being under the same roof, able to have productive discussions inside and outside the classroom. As such, the Staff College in Palace Court, Bayswater, provided accommodation for twenty-four residents – initially young administrators already inside the health service. The central two-year course included lectures on subjects such as the structure of the NHS and its historical background, committee work, personnel issues, public relations, hospital law, finance, and the organisation of services such as catering, supplies, and engineering. The formalisation and assimilation of this kind of knowledge was part of the ongoing process of professionalisation and the broader emotional and philosophical engagement inherent to it, but practical experiences and the development of collective identities were particularly important. As in the US, theoretical knowledge was supplemented by visits to hospitals and health departments, where the trainees saw how things worked in real time. Once again, there were crossovers with physicians working in the same environment, though the result was perhaps closer to the encouragement of empathy inherent in the approach later outlined by Jodi Halpern, rather than the traditional detached concern for medics advocated by Renée Fox and Harold Leif.^43^ Administrators would need to be emotionally engaged, but in the right kinds of ways.

As such, emphasis was placed on the role of the Staff College itself as “a meeting ground for all those — not only practising hospital administrators, officials, and members of governing bodies, but men and women outside the hospital field who have experience and interest in the social life of the community.”^44^ This was important because it was the kind of thing that trainees would be expected to recreate once they returned to or moved into the hospital field. The aim was to “create a congenial and sympathetic environment in which the many skilled services and facilities for investigating and treating illness and accident may be brought to bear upon the patient’s needs, quickly, efficiently, and with economy consistent with those needs.”^45^ If administrators were visible, accessible, and able to help people, then they would “nourish” a “happy hospital.”^46^ Happiness in this form has been understood as a modern development – the idea of a positive environment in which everyone is content and able to satisfactorily perform a role and meet their own needs, but also one which has to be actively pursued and and maintained over time.^47^

Discussions of hospital administration during this period often centred on these two complementary elements – the character of the successful administrator and the emotional intelligence inherent in the role. In his 1966 guide to the field, Geoffrey A. Robinson, then Secretary and Treasurer to the Board of Governors of the National Hospital for Nervous Diseases in London, highlighted the importance of “wise judgment, humanity and the ability to carry responsibility,” as well as being able to “delegate work, inspire others to give of their best and keep a sense of perspective.”^48^ Interestingly however, Robinson also sounded a note of caution: “Let us not pretend that is ever possible to achieve perfection, nor that there will never be occasions when feelings run pretty high. But as long as views are honestly held and expressed, difficulties can be aired, explored and resolved without upsetting the proper atmosphere of the hospital.”^49^

In the US this question had been addressed by Rodney F. White of the Sloan Institute of Hospital Administration at Cornell University, who argued that:

In some cases the problem lies in an administrator’s desire to run a happy shop in which everything runs smoothly and there is a minimum of conflict. Although this certainly is desirable in one sense, the periodic occurrence of conflict in complex organizations is almost inevitable and those organizations that lack it are either superbly managed or are merely existing rather than growing or developing.^50^

It was often recognised therefore that hospital administration was a challenging career – one that should not be entered into lightly. The stakes were high. Only those of particular ability were likely to be able to do it well. Nonetheless, this meant that it could also be uniquely rewarding. The emotional investment required was significant but ultimately worth it. According to Constable, “With all the uncertainties and especially in stringent times the frustrations and delays, the life is never dull, and at its best, as a piece of social engineering, is a challenge to the creative urge and the desire to render service to our fellow-men in a way that few other careers can match.”^51^

## The National Administrative Training Scheme

It was widely recognised that the King’s Fund had taken an important step in the right direction by setting up the Staff College. Sir George Godber, Chief Medical Officer between 1960 and 1973, later argued that “if they had not shown the way, it is unlikely that the Health Service would have gotten off the ground nearly so quickly.”^52^ Nonetheless, inside the Ministry of Health, officials felt that it was necessary to go further, particularly in terms of attracting more university graduates into the hospital service, and discussions about establishing a formal national training scheme began.^53^ In 1951 Godber, when Deputy Chief Medical Officer, and senior civil servant John Pater undertook their own research trip to the US and Canada, just as the King’s Fund had done a year earlier. Their itinerary was more extensive, taking in thirty hospitals and ten universities, but many of the key observations were the same. In particular it was seen again that, despite the readily apparent differences between the two systems, many of the pressures facing administrators – balancing the budget, managing admissions, and maintaining staff relationships – were the same, and when compared to the US experience, on the job training like that still common in British hospitals was inadequate. This was the case in terms of technical skill, but also in terms of gaining the necessary wider perspective: “The mental training, the breadth and depth of outlook needed by the hospital administrator of the future cannot be satisfactorily given by methods of this kind. What is needed above all is education of the university type, using all the techniques of lectures, seminars, group discussions, field trips, paper work, etc. supplemented by carefully supervised practical training in the hospital itself.”^54^ Godber and Pater were clear that they too saw the character and emotional intelligence of the individual as being fundamentally important. Training was the key to harnessing positive traits and tapping into something deeper: “The objective should be the inculcation of principles not of tricks…training of the leaders of a profession in attitudes of mind and ways of thought…Techniques can be acquired by experience later on; principles must be inculcated at the outset.”^55^

As such, a National Administrative Training Scheme eventually began in 1956, run by the King’s Fund at the Staff College in London and the University of Manchester. A national selection committee was established to judge the applicants and allocate places, with those in the south principally going to the King’s Fund and those in the north to Manchester. Trainees undertook a circuit of both theoretical and practical training in the lecture theatre and across general, specialist and teaching hospitals, and in Hospital Management Committee and Regional Health Board offices, each lasting a number of weeks, as part of a process known as “planned movement.”^56^ Former administrators have frequently highlighted this dimension of the training scheme, which continued for many decades, as being important. The fact that they were able to get this hands-on experience – to see how the different departments worked and talk to different members of staff – gave the trainees something important, which they felt they were able to use in the course of their careers. John Wyn Owen, who began the National Administrative Training Scheme in 1964 and went on to be District Administrator of St Thomas’s health district in London, Director of the NHS in Wales, and Secretary of the Nuffield Trust, recalled:

I learned to make hospital corners and twenty-two beds in very short order. I learned how to be an effective porter … we also had to do the very practical jobs like doing the midnight bed return, filing — trying to find the files, taking them to clinic — hanging on to a leg in theatre while the surgeon was trying to pin it, attending post mortems … we actually understood right the way from the bottom what are the nuts and bolts that make this thing work.^57^

Many trainees have also spoken of the values that were instilled in them. Some came to see administration as a “vocation” or a “calling” – evocative of more character driven or caring and rewarding conceptions of work.^58^ At Manchester the scheme was overseen by the influential Professor of Social Administration Teddy Chester, who had developed an interest in management during his time at the Acton Society Trust, an offshoot of the Joseph Rowntree Reform Trust established by Schuster, which sought to “analyse the implications of the welfare state for liberty and the individual.”^59^ Chester wanted his trainees to be “imaginative, farsighted coordinators of all aspects of the service.”^60^ The implicit aim was to produce an elite set of well-trained individuals who saw themselves as being set on a distinctive career path.^61^ Chester has since been warmly remembered by many former trainees, who saw him as an “inspirational” tutor and mentor – even a “father figure” – who was greatly interested in his students, before and after they joined the health service. Robin Stewart, who began the training scheme in 1958 and went on to be General Manager of the Highland Health Board, among other senior positions in Scotland, recalls that Chester was “Inspirational in the sense of selling, not exactly in the words of one syllable, but just by the general approach and the general atmosphere – ‘You can do the same’. ‘You’re entering a worthwhile career. You can make a success of it, and you will be upholding a job which is of good public service interest to the community.’”^62^

Chester was also well known for his extensive networking. This was often put to good use on behalf of his students, who were encouraged to be confident and critical thinkers. David Robson, who also joined in 1958 and later became the District Administrator in Hereford and Worcester Area Health Authority, among other senior positions, remembers that, “He always used to say to us, all these people I’m going to introduce you to are people who know about things, they influence the service, they’ve been around a long time, you must push them. And, therefore, your behaviour in their company doesn’t have to be reverential, it should be incisive, it should be strong, you should make them possibly feel uncomfortable, he didn’t think it wrong to challenge.”^63^Manchester graduates subsequently came to be known as “Teddy Boys” – or “Teddy’s children, if not boys entirely.” The collective identity of the group was reinforced by the wearing of a special tie and the organising of regular get-togethers. Evening sessions with outside guests, which continued for many years, were affectionately known as “Chester’s tea parties.”^64^ From 1964 all national graduates also had the opportunity to join the October Club, which sought to preserve links between trainees and organise regular meetings and lectures – though some came to feel that it was quite “London-centric” and primarily for the “gentleman” of the King’s Fund. For their own part, Staff College trainees were often aware of their reputation. Owen recalled that “We used to compare and contrast ourselves to the Manchester lot, and you could see why we would look elitist. Morning tea in bed at the Staff College? Silver teapot? You don’t get that in Manchester.”^65^

These kind of collective identities, underpinned by status, respect and a sense of camaraderie, reinforced the established conception of hospital administration and the increasingly professional outlook and emotional engagement at the heart of it. It was also important that civil servants made a conscious effort to recruit the best graduates and compete with the more established career routes such as entry into the civil service. Undergraduates at college careers evenings such as those organised by the Oxford and Cambridge University Appointments Boards would be able to speak to experienced Hospital Board and Hospital Management Committee Secretaries and Deputy Secretaries who promoted the national training scheme. According to Bob Nicholls, who went on to be Chief Executive of Oxford Regional Health Authority and part of the NHS Management Executive, a career in hospital administration was made to seem attractive: “The major influence was the fact that the person who came to do the Cook’s Tour for the NHS sold the national management graduate training scheme and in particular … he sold Teddy Chester.”^66^ Similarly, Stewart recalls, “The civil service was generally the kind of idea I had, and I was entered for the civil service exams. But at the Cambridge University Appointments Board, I was told about alternatives which were similar. One was the National Coal Board I remember, one was the BBC and one was the Hospital Training Scheme, which I’d never heard of before.”^67^

As the National Administrative Training Scheme steadily expanded in subsequent years, emphasis continued to be placed on these kinds of opportunities and the personal qualities required of a good administrator. A King’s Fund pamphlet from the 1960s described how:

There are few careers in the public service which provide such a wide variety of experience, ranging from committee and office work to personal contact with staff of all kinds, and with patients and their relatives. It is a career in which personal qualities count for a good deal. The type of person needed is one possessing a wide humanity, and capable of carrying considerable responsibility and exhibiting a balanced judgement. He must be able to get on with people of all kinds, to see broad problems in their proper perspective, to giver personal attention to detail when it’s needed, and to inspire a respect in those with whom he works.^68^

The extent to which, as a result, the national trainees might have been young, ambitious and had slightly heightened expectations, was sometimes clear once they then entered the hospital field. Maurice Naylor, who studied under Teddy Chester and later became Secretary of Sheffield Regional Hospital Board and Regional Administrator of Trent Regional Health Authority, recalled of one senior colleague, “Nice man, but he wouldn’t retire, and I kept nudging him. There was nothing for me to do being Deputy to this man.”^69^ When the expected outlook was absent, the ideal of the “happy hospital” was less likely to be realised.

Ann Cartwright of the Institute of Community Studies (established in 1953 by Michael Young) also cautioned administrators against falling back into old-fashioned “attitudes of condescension” which could easily begin to resemble undesirable “legacies from the era of charity and custodial care.”^70^ Rosemary Stevens (then Rosemary Wallace), who went on to have a successful career in academia, had previously been a hospital administrator and kept a diary during her first placement as a national trainee in Sheffield in late 1957 and early 1958 – the kind of primary source that is now hugely valuable to historians. She recorded the “surely fictional story of the official who went into the hospital waiting room and asked those sitting there, ‘Who’s for death and who’s for admission?’”^71^

Although long-serving administrators with established ways of working could sometimes be a source of frustration, it was also possible for trainees to appreciate when a senior colleague had been doing a good job and shared the right kind of perspective. For example, Stevens recognised that some colleagues had already been achieving the kinds of things that she would aim for herself. As she wrote of one hospital secretary, “His policy is one of ‘kindly benevolence’ and ‘jolly-good fellowship.’ He radiates good-will and calls himself ‘everybody’s slave.’ He sees himself as a liaison officer between different groups of staff, rather than a decision-maker…So far, this stance seems to have served him well. In terms of the staff, the hospital seems a happy place.”^72^ As discussed, there was often a high degree of continuity inside the NHS despite wider changes, and the central tenets of character and emotional intelligence in support of efficiency were often in place before being drawn out further and inculcated by subsequent professionalisation. Ken Jarrold, who began the National Administrative Training Scheme in 1969 and went on to be Regional General Manager of Wessex RHA and NHS Deputy Chief Executive, recounts a story in his memoir in which as a young administrator he asked a senior colleague for a hole in his office carpet to be repaired:

Jack asked me what I would do if a ward sister came into my office (something that happened a lot) and asked me for a new carpet for the ward dayroom and I had to say no because we could not afford it. What, he asked, would the ward sister think when she looked down and saw my new carpet?.... If on the other hand, I asked the sister to look down and inspect the hole in my carpet, I would be able to say, “Sister, look at the state of my carpet. Times are hard.” The sister would go back to her colleagues and ask if they had seen the state of the poor deputy superintendent’s carpet and tell them that she was content to make do with the day room carpet for another year.^73^

We can see therefore some that a hospital administrator with the right kind of approach and requisite degree of emotional intelligence would be able to create a positive and collegiate environment whilst also ensuring overall efficiency. The two would go hand in hand.

## Conclusion

By examining the history of modern hospital administration and beginning to draw out the place of emotion, we can shine an interesting new light on the relationship between professional identity and emotional landscapes in the healthcare field. In doing so, this article complements recent studies which have sought to assess the place of emotions in the life and work of medical professionals and the wider construction of the hospital environment. There is real potential for future work in the history of emotions which draws on the formative experiences of hospital administrators. Researchers are likely to find fertile ground and plentiful material when they consider in more detail issues such as the sex, gender roles, age, class, race, and ethnicity of individuals and groups for example, and the emotional stories of the institutions in which they worked.

The immediate focus of this article has been the broader emotional and philosophical investment that many hospital administrators made in their work. In both the US and Britain from the early decades of twentieth century, amidst rapid change in the practice and provision of health services and shifts towards medical corporatism, a collective sense of professional identity emerged, often underpinned by the conceptualisation and utilisation of emotion. Four key thematic points can help us to understand this. First, the education and trainings undertaken by administrators were central to the development of these kinds of collective and constructive patterns and speak to the co-constitutive nature of formal training and the formation of professional identities.^74^ Of particular importance to many administrators was the structured practical experience that they were able to gain as part of postgraduate courses in the US from the 1930s and the National Administrative Training Scheme first established in Britain in 1956, including the opportunity to visit hospitals, see how they worked in real time, and begin to understand the perspectives of different members of staff. This helped to prepare them for the varied emotional world of the hospital, but it also helped to inculcate a historically situated appreciation of the need for efficiency – an important part of the wider emotional investment.

Second, as a result, the personal qualities required of a good administrator became central to conceptions of the role. References to trainees needing “initiative” and “imagination” but also “tact” and “humanity” were common. When putting what they had learned into practice, a good administrator would seek to create a positive and collegial environment, balancing the needs of patients with those of medical professionals, whilst ensuring overall economy. The aim was a “happy hospital” – one that treated patients compassionately but efficiently.

Third, this conception of hospital administration, and the emotional engagement at the heart of it, was often reinforced through the development and maintenance of collective identities at an institutional and an individual level. Many administrators felt strongly about their own hospital or local area, or their own healthcare system, but this dynamic seems to have been particularly tangible in terms of feelings of camaraderie, respect for influential tutors and mentors, and pride in the values that were instilled in them, as part of those kind of postgraduate programmes and training courses.

Fourth, it is striking that such developments in Britain were influenced by best practice in the US where training patterns were already well established and hospital administration was more accepted as a profession. Though this process might best be understood as a providing a catalyst for the further drawing out of established beliefs and ways of working in Britain rather than an abstract transfer of ideas and practices across the Atlantic, there was a distinct Anglo-American dimension to the development of hospital administration that it is important to appreciate.

## Footnotes

1
https://www.rcn.org.uk/magazines/bulletin/2020/feb/history-of-emotions-in-nursing.

2 Agnes Arnold-Forster, “Operating with Feeling: Emotions in Contemporary Surgery,” *Bulletin of the Royal College of Surgeons* 100 (2018): 13; Agnes Arnold-Forster, “‘A Small Cemetery’: Death and Dying in the Contemporary British Operating Theatre,” *Medical Humanities* 46 (2020): 278–287.

3
https://academic.oup.com/Shm/pages/senses_virtual_issue_introduction; https://hospitalsenses.co.uk/.

4 Victoria Bates, “‘Humanizing’ Healthcare Environments: Architecture, Art and Design in Modern Hospitals,” *Design for Health* 2 (2018): 5-19.

5 Rosemary [Wallace] Stevens, *Observations of a National Health Service Hospital: An Administrative Trainee’s Diary, October 1957-January 1958*. Unpublished manuscript, 16.

6 Interview with John Wyn Owen, May 2016.

7 Rob Boddice, *The History of Emotions* (Manchester: Manchester University Press, 2017); Jan Plamper, *The History of Emotions: An Introduction* (Oxford: Oxford University Press, 2017); Susan J. Matt and Peter N. Stearns, *Doing Emotions History* (Urbana: University of Illinois Press, 2014); Katie Barclay, Sharon Crozier-De Rosa, and Peter N. Stearns, ed., *Sources for the History of Emotions: A Guide* (Abingdon: Routledge, 2020); Barbara H. Rosenwein, “Problems and Methods in the History of Emotions,” *Passions in Context: Journal of the History and Philosophy of the Emotions* 1:1 (2010), https://www.passionsincontext.de/.

8 William M. Reddy, *The Navigation of Feeling: A Framework for the History of Emotions* (Cambridge: Cambridge University Press, 2001), 330.

9 Arlie R. Hochschild, *The Managed Heart: Commercialization of Human Feeling* (Berkely: University of California Press, 2012); Sara Ahmed, *The Cultural Politics of Emotion* (Edinburgh: Edinburgh University Press, 2014).

10 John Kirk and Christine Wall, *Work and Identity: Historical and Cultural Contexts* (Basingstoke: Palgrave Macmillan, 2011).

11
*Administration, Management and Training in the National Health Service: The Contribution of Professor Teddy Cheste*r, held 14 March 2019 at the University of Manchester, published by the Department of Public Health and Policy, University of Liverpool, 2020: https://www.liverpool.ac.uk/population-health-sciences/departments/public-health-and-policy/research-themes/governance-of-health/witness-seminars/posts/amtteddychester/.

12 John R. McGibony, *Principles of Hospital Administration* (New York: Putnam, 1952), 3.

13 Steve Sturdy and Roger Cooter, “Science, Scientific Management, and the Transformation of Medicine in Britain, c.1870-1950,” *History of Science* 36 (1998): 423-425.

14 Charles E. Rosenburg, *The Care of Strangers: The Rise of America’s Hospital System* (Baltimore: Johns Hopkins University Press, 1995), 42.

15 Morris J. Vogel, “Managing Medicine: Creating a Profession of Hospital Administration in the United States,” in *The Hospital in History*, ed. Lindsay Granshaw and Roy Porter (London: Routledge, 1989), 244.

16 Rosemary Stevens, *In Sickness and in Wealth: American Hospitals in the Twentieth Century* (Baltimore: Johns Hopkins University Press, 1999), 156.

17 Vogel, “Managing Medicine,” 248.

18 Stevens, *In Sickness and in Wealth*, 72.

19 McGibony, *Principles of Hospital Administration*, 4.

20 Boddice, *History of Emotions*, 140.

21 John H. Warner, “The Humanising Power of Medical History: Responses to Biomedicine in the 20th Century United States,” *Medical Humanities* 37 (2011): 91-96.

22 McGibbony, *Principles of Hospital Administration*, 184-187.

23 Ibid., 186.

24 Boddice, *History of Emotions*, 57.

25 A.R. Vargas, “Development of Formal Education for Hospital Administration,” in *Modern Concepts of Hospital Administration*, ed. Joseph K. Owen (Philadelphia: Saunders, 1962), 728.

26 Stevens, *In Sickness and in Wealth*, 158.

27 George F. Billington, *Cases in Hospital Administration* (New York: Columbia University, 1959), 14.

28 Gerhard Hartman, “Graduate Education in Hospital Administration, 1934-37,” *Journal of Business of the University of Chicago* 11 (1938): 375.

29 Billington, *Cases*, 4.

30 Ibid.

31 John Pantall, in *Administration, Management and Training in the National Health Service: The Contribution of Professor Teddy Cheste*r, Transcript of a Witness Seminar, University of Manchester, 14 March 2019, 19, https://pcwww.liv.ac.uk/~gjones89/TeddyChesterWitnessSeminarTranscriptFinal.pdf.

32 The National Archives, MH/55/1435.

33 Frank Hart and A.J. Waldegrave, *A Study of Hospital Administration* (London: Stevens, 1948), 27.

34 Martin Gorsky, John Mohan, and Martin Powell, “Hospitals and the Public Sphere: Contribution and Participation before the National Health Service,” in *Medicine, Health and the Public Sphere in Britain, 1600-2000*, ed. Steve Sturdy (Abingdon: Routledge, 2002); George C. Gosling, *Payment and Philanthropy in British Healthcare, 1918-48* (Manchester: Manchester University Press, 2017).

35 London Metropolitan Archives, A/KE/G/01/11/005.

36 Hart and Waldegrave, *Study of Hospital Administration*, viii.

37 Ibid.

38 Ibid, 177.

39
*Hospitals and the State: Hospital Organisation and Administration Under the National Health Service – Creative Leadership in a State Service: A General Survey* (London: Acton Society Trust, 1959), 59.

40 Victoria. Bates, “Yesterday’s Doctors: The Human Aspects of Medical Education in Britain,” *Medical History* 61 (2017): 64.

41 C.R. Jolly, “Administrative and Clerical Staffing,” in *Modern Hospital Management*, ed. J.F. Milne and N.W. Chaplin (London: Institute of Hospital Administrators, 1969), 206.

42 Andrew Seaton, “The Gospel of Wealth and the National Health: The Rockefeller Foundation and Social Medicine in Britain’s NHS, 1945-60,” *Bulletin of the History of Medicine* 94 (2020): 91-124.

43 Jodi Halpern, *From Detached Concern to Empathy: Humanizing Medical Practice* (Oxford: Oxford University Press, 2011); Harold I. Lief and Renée C. Fox, “Training for ‘Detached Concern’ in Medical Students,” in *The Psychological Basis of Medical Practice*, ed. Harold I. Lief, Victor F. Lief, and Nina R. Lief (New York: Harper & Row, 1963), 12-35; N.R. Angoff, “Making a Place for Emotions in Medicine,” *Yale Journal of Health Policy, Law, and Ethics* 2 (2002): 447-454.

44 LMA, A/KE/G/01/11/002, “Hospital Administration and the Contribution of the King’s Fund.”

45 Ibid.

46 Ibid.

47 Rosenwein, “Problems and Methods”; Darrin M. McMahon, *Happiness: A History* (New York: Grove Press, 2006).

48 Geoffrey A. Robinson, *Hospital Administration* (London: Butterworths, 1966), 57.

49 Ibid., 551.

50 Rodney F. White, “Current Trends in Administration,” in *Modern Concepts of Hospital Administration*, 764.

51 P. Constable, *Training in Hospital Administration* (London: King’s Fund, 1950), 24.

52
*The British National Health Service: Conversations with Sir George Godber* (Bethesda, MD: US Department of Health, Education, and Welfare, 1976), 109.

53 TNA, MH 123/79.

54 Ibid.

55 Ibid.

56 Ibid., “Recruitment and Post-Entry Training Content: Content of Training Course for Selected Entrants.”

57 Interview with Owen.

58 Sarah Chaney, “Before Compassion: Sympathy, Tact and the History of the Ideal Nurse,” *Medical Humanities* 47 (2021): 475-484.

59
https://www.rowntreesociety.org.uk/history/rowntree-a-z/joseph-rowntree-reform-trust/.

60 Stephanie Snow, “‘I’ve Never Found Doctors to Be a Difficult Bunch’: Doctors, Managers and NHS Reorganisations in Manchester and Salford, 1948-2007,” *Medical History* 57 (2013): 65-86.

61 Ibid.

62 Robin Stewart, in *Administration, Management and Training in the National Health Service*, 20.

63 David Robson, in *Administration, Management and Training in the National Health Service*, 17.

64 Kieran Walshe, in *Administration, Management and Training in the National Health Service*, 51.

65 Interview with Owen.

66 Interview with Bob Nicholls, May 2017.

67 Robin Stewart, in *Administration, Management and Training in the National Health Service*, 20.

68 Ibid.

69 Interview with Maurice Naylor, October 2016.

70 Ann Cartwright, *Human Relations and Hospital Care* (London: Routledge, 1964), 203.

71 Stevens, *Observations of a National Health Service Hospital*, 9.

72 Ibid., 6.

73 Ken Jarrold, *Other People’s Shoes: 40 Questions for Leaders and Managers* (Stockton-on-Tees: CreateSpace Independent Publishing Platform, 2018).

74 Rosalia Cascón-Pereira and Jerry Hallier, “Getting That Certain Feeling: The Role of Emotions in the Meaning, Construction and Enactment of Doctor Managers’ Identities,” *British Journal of Management* 23 (2011): 130-144; Sumati Ahuja, Helena Heizmann, and Stewart Clegg, “Emotions and Identity Work: Emotions as Discursive Resources in the Constitution of Junior Professionals’ Identities,” *Human Relations* 72 (2018): 988-1009; Kate E. O’Connor, “’You Choose to Care’: Teachers, Emotions and Professional Identity,” *Teaching and Teacher Education* 24 (2008): 117-126.

